# Initial Experience with Robotic Retropubic Urethropexy Compared to Open Retropubic Urethropexy

**DOI:** 10.1155/2013/315680

**Published:** 2013-09-08

**Authors:** Pooja R. Patel, Mostafa A. Borahay, Audrey R. Puentes, Ana M. Rodriguez, Jessica Delaisse, Gokhan S. Kilic

**Affiliations:** Department of Obstetrics and Gynecology, The University of Texas Medical Branch in Galveston, 301 University Boulevard, Galveston, TX 77555-0587, USA

## Abstract

*Study Objective*. To compare the clinical outcomes of robotic retropubic urethropexy versus open retropubic urethropexy. *Design*. Retrospective case-control study (II-2). *Setting*. University Hospital. *Patients*. All patients who underwent robotic retropubic urethropexy from 1/1/12 to 6/1/12 by a single gynecologic surgeon were included in the case series. The control cases consisted of the last five consecutive open retropubic urethropexies performed by the same surgeon. *Main Results*. A total of 10 patients (5 robotic cases and 5 open cases) were included in this study. Both groups were similar with respect to age, BMI, and obstetrical history. Mean hospital stay length and mean EBL were overall less for robotic cases than for open cases (1.2 days versus 2.6 days; 169 mL versus 300 mL). One of the 5 patients who underwent the open approach and 2 of the 5 patients who underwent the robotic approach sustained a minor intraoperative complication. All but one patient from each group experienced resolution of incontinence after the procedure. Two of the patients who underwent the open approach had postoperative complications. *Conclusions*. Robotic retropubic urethropexy may be a feasible alternative to open retropubic urethropexy. A larger study is necessary to support our observations.

## 1. Introduction

Minimally invasive surgery has gained tremendous popularity over the recent decades. This popularity is due to several advantages associated with robotic surgery versus laparotomy, which include magnification resulting in better visualization, decreased blood loss and need for blood transfusions, shorter hospital stay and postoperative recovery, decreased postoperative pain, and decreased risk of infections [1–6]. Even more recently, robotic surgery has gained popularity due to improved precision and surgeon comfort when compared to laparoscopy. As experience and comfort with robotic surgery have increased, the robot is being used now for more comples surgeries, including urogynecologic procedures such as the retropubic urethropexies (i.e., Burch colposuspensions).

 Pelvic organ prolapse (POP) is becoming a more prominent problem, especially given the aging US population. The estimated lifetime risk of surgery for stress urinary incontinence or POP is 11%; however, this is likely an underestimate of the general population as it is based on hospital data [6]. 

 The current literature surprisingly lacks information on experiences with robotic retropubic urethropexy in comparison to retropubic urethropexy associated with laparotomy (i.e., open retropubic urethropexy). Partin et al. reported on experiences with 2 robotic retropubic urethropexies as part of a cohort including 17 patients undergoing robotic-assisted urologic procedures [7]. Payne and Dauterive described results in 8 patients treated with robotic Burch procedures at the time of robotic hysterectomy; however, as their aim was to compare perioperative outcomes between patients undergoing total laparoscopic hysterectomy and robotic hysterectomy, they did not report specifically on outcomes of the urethropexy portion of the procedure nor did they compare this to patients who underwent open retropubic urethropexy [8]. Khan et al. described 2 cases of successful retropubic urethropexies [9]. We were unable to find any case series in the literature pertaining to retropubic urethropexies via robotic approach.

The objective of our study was to report on experiences with both open and robotic retropubic urethropexies performed by the same surgeon. In this case series we report on perioperative and postoperative outcomes.

## 2. Patients and Methods

 After Institutional Review Board approval from the University of Texas Medical Branch in Galveston, the medical records of all patients who underwent robotic retropubic urethropexy from 1/1/12 to 6/1/12 by a single gynecologic surgeon at the John Sealy Hospital at the University of Texas, Medical Branch in Galveston, were reviewed retrospectively. The control cases consisted of the last five consecutive open retropubic urethropexies prior to 1/1/12, performed by the same surgeon. Prior to this case series the surgeon had performed over 100 robotic cases and had at least 10 years of experience performing open retropubic urethropexies.

Data collected and analyzed for this study included age, race/ethnicity, body mass index (BMI), number of spontaneous vaginal deliveries (SVDs), gravidity, parity, history of prior abdominopelvic surgery, details regarding incontinence prior to and after the procedure, length of postoperative hospital stay, intraoperative complications, estimated blood loss (EBL), and postoperative complications. Intraoperative complications were defined as cystotomy, suture penetrating bladder wall, vascular injury, injury to bowel, or injury to the ureters. 

 Robotic and open retropubic urethropexies were performed by first distending the bladder with 500 mL sterile normal saline via the Foley catheter for easier identification of the plane between the bladder and the upper pelvic wall. Next, the areolar tissue between the pubic bone and the bladder was dissected, creating the space of Retzius and allowing for identification of Cooper's ligament. The urethrovesical junction was subsequently identified with a finger in the vagina and manipulation of the Foley catheter. The periurethral tissue was then suspended to Cooper's ligament using 2/0 polypropylene stitches bilaterally, resulting in a total of four stitches: two stitches on each side, lateral to the urethrovesical junction (UVJ), and 2 stitches on each side, lateral to the proximal third of the urethra. The finger in the vagina was used to apply upward pressure in order to identify the appropriate areas in pelvic floor. Areas exposed were grasped with an intra-abdominal grasper to guide the suture placement. Cystoscopy was performed after each case.

Postoperative data was retrospectively collected from the 3-month postoperative clinic visit.

## 3. Results

A total of 10 patients were included in this study, 5 patients who underwent open retropubic urethropexy and 5 who underwent robotic retropubic urethropexy. Demographic data is summarized in Table 1. Ages (years) ranged from 35 to 50 in the open group and 41 to 61 in the robotic group (mean age = 44.2 and 46.6, resp.). The majority (3 out of 5) patients were African American in the open group and Caucasian (3 out of 5) in the robotic group. BMI (kg/m^2^) ranged from 28 to 33 in the open group and 28 to 37 in the robotic group (mean BMI = 30.5 and 31.2, resp.). In both groups, majority of patients did not smoke (3 out of 5 for the open group; 4 out of 5 for the robotic group). All but one patient in both the open and robotic groups had at least 2 SVDs; the remaining patient in each group had two cesarean sections (CS). Regarding surgical history, in the open group, 2 patients had no previous abdomino-pelvic surgery, while one patient had a splenectomy and BTL, another patient had a previous CS, and a third had two previous CS. In the robotic group, 3 patients had no previous abdomino-pelvic surgery (one of these had a hysteroscopic myomectomy), one patient had two previous CS, and one patient had a laparoscopic cholecystectomy and gastric bypass. None of the patients had previous surgery for POP. Mean age, BMI, spontaneous vaginal deliveries (SVDs), gravidity, and parity are also provided in this table. All patients underwent simple cystometry to confirm stress urinary incontinence before surgery. 

Hospital-related data are summarized in Table 2. The average hospital stay for the patients in the open group was 2.6 days, while average hospital stay for patients in the robotic group was 1.2 days with all but one patient going home on the day after surgery. EBL ranged from 200 mL to 600 mL (mean = 300 mL) in the open group and from 20 mL to 400 mL (mean = 169 mL) in the robotic group. Average time to complete the retropubic urethropexy in the open group was 27 minutes, while average time to complete the retropubic urethropexy in the robotic group was 36 minutes. Regarding intraoperative complications, one of the patients (O1) who underwent open retropubic urethropexy was noted on cystoscopy to have a suture penetrating the bladder wall. Of the patients who underwent robotic retropubic urethropexy, one patient (R1) had a right ureteral stent placed intraoperatively due to concern for excessive right pelvic wall dissection to prevent possible microperforation of the ureter, which was removed 6 weeks postoperatively. A different patient (R2) sustained a minor injury to the right uterine artery that required a vascular clip. Of note, both robotic intraoperative complications did not occur during the retropubic urethropexy portion of the surgeries.

 Postoperative outcome with regard to incontinence and postoperative complications is presented in Table 3. All patients reported clinical incontinence prior to surgery. One open case (O1) and one robotic case (R5) reported postoperative incontinence. With regard to postoperative complications, one of the patients (O1) who underwent open retropubic urethropexy experienced an abdominal incision wound separation on postoperative day 10 which had healed completely 4 months post-operatively. Another patient who underwent open retropubic urethropexy experienced fevers (clinically attributed to atelectasis) in the hospital which resolved prior to discharge (postoperative day 4). None of the patients who underwent robotic retropubic urethropexy experienced a postoperative complication.

## 4. Discussion

 In this case series we present our initial experience with robotic retropubic urethropexies. We also present the last five open retropubic urethropexy cases the same surgeon performed prior to adopting the robotic approach. We assume the surgeon had completed the robotic learning curve as he had completed over 100 robotic cases prior to this case series. Statistical analysis on demographic data was not performed given the small sample size of both open and robotic groups. However, the mean age, BMI, SVDs, gravidity, and parity are quite similar between the two groups, allowing for a better comparison when looking at surgical data and outcomes. We do note, however, that the majority of the patients who underwent the robotic approach were Caucasian (3 out of 5), whereas the majority of the patients who underwent the open approach were African American (3 out of 5). When looking at the data with regard to hospital days and estimate blood loss, the well-known benefits of a robotic approach versus an open approach are obvious. Although two robotic patients sustained intraoperative complications, the extent of these complications was small as one patient (R1) went home with a prophylactic ureteral stent on postoperative day 2 (which was subsequently removed without sequelae), and the other robotic patient (R2) whose surgery required a vascular clip had total EBL which was only 100 cc more than the average of the open retropubic urethropexy. In addition, all intraoperative complications did not occur during the retropubic urethropexy portion of the surgery. With regard to the operative time, the open approach was faster to complete than the robotic approach. However, it is important to note that the robotic cases were the first five performed by the surgeon, and, therefore, time to completion will likely be less with more experience. With regard to the postoperative complications, none of the patients who underwent robotic retropubic urethropexy sustained a postoperative complication, whereas among the open retropubic urethropexies, the patient who sustained a wound separation required more frequent postoperative visits and decreased quality of life during the healing process. Finally, the fact that one patient from each complained of postoperative incontinence suggests equal symptomatic outcome for a robotic approach when compared to an open approach.

 This case series shows that a robotic approach to retropubic urethropexy may be equal to if not better than an open approach with regard to perioperative and postoperative outcomes. In addition to outcomes, a robotic approach allows for better visualization of the retropubic region and better ergonomics for the surgeon. A larger scale comparison is warranted to confirm our findings. Nevertheless, to the best of our knowledge, this is the largest retrospective review of patients who have undergone robotic retropubic urethropexy and the only review that compares outcomes to open cases performed by a single, robotically experienced surgeon. 

## Figures and Tables

**Table 1:** Demographic information for each retropubic urethropexy case.

	Open						Robotic					
	O1	O2	O3	O4	O5	Mean	R1	R2	R3	R4	R5	Mean
Age	48	44	44	35	50	44.2	41	48	41	42	61	46.6
Race	AA	AA	H	AA	C	—	C	C	H	H	C	—
BMI	32.95	29.0	28.7	29.1	33.0	30.5	29.1	37.9	28.6	32.0	28.3	31.2
Smoking	Yes	No	No	No	Yes	—	Yes	no	No	No	No	—
SVDs	2	2	3	2	0	1.8	3	4	0	2	2	2.2
Gravidity	3	2	3	3	2	2.6	3	5	2	2	2	2.8
Parity	2	2	3	3	2	2.4	3	4	2	2	2	2.6
Previous abdominopelvic surgery	No	Splenectomy, BTL	No	c/s × 1	c/s × 2	—	Laparoscopic cholecystectomy; gastric bypass	Hysteroscopic myomectomy	c/s × 2	No	No	—

H: Hispanic.

AA: African American.

C: Caucasian.

A: Asian.

**Table 2:** Intraoperative details for each retropubic urethropexy case.

	Open						Robotic					
	O1	O2	O3	O4	O5	Mean	R1	R2	R3	R4	R5	Mean
Concomitant surgeries	TAH/BSO/APR	None	TAH	TAH/BSO/Halban culdoplasty	TAH/BSO	—	Robotic hysterectomy	Robotic hysterectomy/robotic Halban culdoplasty/sphincteroplasty/PR/perineorrhaphy	Robotic hysterectomy	Robotic Halban culdoplasty/robotic uterosacral ligament suspension	Robotic myomectomy/robotic hysterectomy/robotic BSO/robotic sacrocolpopexy	—
Operative time for urethropexy (dissection/suture)*	25 (13/12)	25 (11/14)	20 (9/11)	32 (15/17)	33 (14/19)	27 (12/15)	45 (20/25)	36 (17/19)	25 (15/10)	22 (10/12)	55 (30/25)	36 (18/18)
Intraoperative complications	Yes**	No	No	No	No	—	Yes***	Yes****	No	No	No	
EBL (cc)	300	600	200	200	200	300	100	400	250	20	75	169
Hospital days	2	4	2	2	3	2.6	1	2	1	1	1	1.2

TAH: Total abdominal hysterectomy; BSO: bilateral salpingoophorectomy; APR: anterior posterior repair.

*Time taken to perform the retropubic urethropexy portion of the surgery. First number is the dissection time in minutes. Second number is suturing time in minutes.

**Suture noted in bladder on cystoscopy.

***Right ureteral stent placed due to concern for excessive electrocautery use.

****Vascular clip used on right uterine artery due to bleeding.

**Table 3:** 

	Open					Robotic				
	O1	O2	O3	O4	O5	R1	R2	R3	R4	R5
Postoperative incontinence	Yes	No	No	No	No	No	No	No	No	Yes
Postoperative complications	Yes^*γ*^	Yes^*δ*^	No	No	No	No	No	No	No	No

^*γ*^Wound separation.

^*δ*^Postoperative fever with clinical diagnosis of atelectasis.
